# 

**Published:** 1888-06

**Authors:** 


					﻿m. VIII.
JUNE, 1888.
No. 6.
IPHE
fiOWKEOPATHlC pHY^lClAjI,
A Monthly Journal of Homoeopathic Materia Medica
and Clinical Medicine.
" He is a freeman whom truth makes free, and all are slaves beside.”
“Seek the Truth: come whence it may, cost what it will.”
EDITED AND PUBLISHED BY
Edmund j. lee, m. d.,
AND
WALTER M. JAMES, M. D.
PHILADELPHIA:
No. 1123 SPRUCE STREET.
X.O2ST3DOTT1
ALFRED HEATH & CO., Sole Agents fok Great Britain,
114 Ebury Street, Eaton Square.
j8S* Yearly Subscription, $2.50, invariably in advance. Single numbers, 25 cts.
Entered at the Philadelphia Poet-Office a* Second-Class Mall Matter.
				

